# Assessment of Indicators of Left Ventricular Performance Obtained by Tissue Doppler Imaging in Prematurely Born Neonates

**DOI:** 10.3390/jcdd9110364

**Published:** 2022-10-23

**Authors:** Nenad Barišić

**Affiliations:** 1 Faculty of Medicine, University of Novi Sad, 21000 Novi Sad, Serbia; nenad.barisic@mf.uns.ac.rs; Tel.: +381-64-2115233; 2 Institute For Child and Youth Healthcare of Vojvodina, 21000 Novi Sad, Serbia

**Keywords:** neonatology, premature newborns, tissue Doppler, pwTDI, echocardiography

## Abstract

Introduction: Tissue Doppler imaging techniques (pulsed-wave TDI (pwTDI) and color-coded TDI (cTDI)) allow for the assessment of myocardial performance during the cardiac cycle. The application of such techniques in neonatology is sporadic and poorly studied. Objective: The objective of the present study was to determine average values of pwTDI indicators of left ventricular performance (maximum systolic velocity of the mitral annulus (s’), maximum velocity in early diastole (e’) and maximum velocity in late diastole (a’)) and to examine their dynamics in prematurely born newborns in the first week of life. Methods: Prematurely born newborns of postnatal age up to 7 days were divided by gestational age into Group1 (<28 weeks) and Group 2 (≥28 weeks). Standard pwTDI parameters (s’, e’ and a’) were measured, compared between the groups and correlated with gestational and postnatal age, as well as application of respiratory support. Results: Fifty subjects were included (Group 1: 24; Group 2: 26). Average values of parameters s’, e’ and a’ were: Group 1: 4.06 ± 0.78 cm/s, 3.71 ± 0.40 cm/s and 3.98 ± 1.06 cm/s, respectively; Group 2: 4.18 ± 1.22 cm/s, 4.68 ± 1.04 cm/s and 4.12 ± 0.94 cm/s, respectively. Values of parameter e’ differed significantly between groups (*p* = 0.001) and strongly correlated with gestational age (*p* = 0, Pearson’s R = 0.88). There was no significant difference between groups for parameters s’ and a’ (*p* = 0.42 and 0.31, respectively). The values of s’, e’ and a’ did not differ between patients with an without respiratory support. Conclusion: Parameter e’ depends on gestational age, whereas parameters s’ and a’ are independent of gestational age. pwTDI indicators do not change during the first week of life, nor are all robust to hemodynamic circumstances caused by invasive/non-invasive respiratory support.

## 1. Introduction

The increasing use of echocardiographic techniques in neonatal intensive care units (NICU) has resulted in significant progress in the treatment of severely ill neonates in the last ten years, enabling more accurate and rapid assessment of the hemodynamic status of these fragile patients, in addition to introducing the possibility of individually tailored therapy for each patient. There is a current worldwide trend of intensive care pediatricians and neonatologists being trained to apply echocardiographic techniques, enabling the use of data obtained in everyday practice for clinical decision making [1].

No echocardiographic index of cardiac function can assess the contractility of the myocardium per se. Ventricular loading conditions, which can vary in prematurely born neonates for a number of reasons, affect all cardiac function indices. Traditional echocardiographic indicators of cardiac function commonly used in neonatology, such as fractional shortening (FS) or ejection fraction (EF), are subject many shortcomings and are not sufficiently accurate to assess cardiac function in preterm infants [2,3]. Tissue Doppler imaging (TDI) techniques allow for the assessment of myocardial performance during different phases of the cardiac cycle. TDI is a quantitative echocardiographic technique that measures the speed of myocardial movements, made technically possible by eliminating high-frequency ultrasound signals generated by blood flow and focusing on low-frequency signals generated by tissue movement. Myocardial performance in the longitudinal direction can be assessed by measuring the velocity of movements of the mitral and tricuspid valve annulus, as well as by measuring the velocity of the base of the interventricular septum (IVS). With TDI, the ventricular systolic function is assessed by measuring maximum systolic velocity (s’ wave). Diastolic function is assessed by measuring two values: maximum velocity in early diastole (e’ wave) and maximum velocity in late diastole (a’ wave).

Two basic TDI techniques are available: pulsed-wave TDI (pwTDI) and color-coded TDI (cTDI). Both techniques enable the measurement of maximum (peak) velocities and time intervals, and cTDI also enables the assessment of the movement of individual myocardial segments during the cardiac cycle. Although the same parameters can be evaluated, the values of parameters obtained by cTDI are about 20% lower than those obtained using pwTDI techniques, so the values of the same parameters obtained using different TDI techniques cannot be compared [3,4]. These echocardiographic techniques are well-studied and widely used in adult cardiology, whereas their use in pediatrics, especially in neonatology, is not routine. Normal values of commonly used TDI indicators of ventricular performance are not well-defined or sufficiently evaluated in prematurely born newborns. In newborns, TDI techniques are most commonly used to assess left and right ventricular function by measuring mitral and tricuspid valve annulus velocities. In general, high maximal velocities of atrioventricular annulus imply better myocardial performance than low values. Some TDI parameters, such as interventricular septum base rate movement, are not used in neonates because movements and performance of the myocardium of neonates, especially during the neonatal transitional period, are significantly affected by variable hemodynamic circumstances in the right and left ventricle (as a consequence of high pulmonary resistance or the presence of patent arterial duct, due to respiratory distress syndrome, etc.). The most commonly used time index that can be determined using TDI techniques is the myocardial performance index (MPI). MPI defines the fraction (percentage, share) of the time duration of isovolumetric phases in relation to the entire ejection period [3].

The aim of this study is to determine the average values of individual echocardiographic parameters most commonly obtained using the pwTDI technique in neonates (maximum systolic velocity of the mitral annulus (s’), maximum velocity in early diastole (e’) and maximum velocity in late diastole (a’)) and examine their dynamics in premature infants in the first week of life, as well as to determine whether gestational age, postnatal age or use of invasive and non-invasive respiratory support affects the values of these echocardiographic indicators.

## 2. Materials and Methods

The study was conducted as a prospective study that included premature infants who were hospitalized in the neonatology service of the regional pediatrics clinic during the period from 1 December 2020 to 31 June 2021.

Criteria for inclusion in the study were premature birth and postnatal age up to 7 days at the time of echocardiographic examination.

Criteria for exclusion from the study were age of more than 7 days, severe intrauterine growth restriction, presence of major congenital anomalies of anomalies of any organ system, presence of hemodynamically significant arterial duct, presence of clinical signs of circulatory failure, inotropic and vasopressor drugs 24 h before echocardiographic examination, signs of severe hypoxia, presence of massive pneumothorax, clinical and radiological signs of massive hemorrhage in the central nervous system and death within 48 h of echocardiographic examination.

Subjects included in the study were classified into two groups according to gestational age (GA): Group 1, comprising prematurely born neonates with GA up to 27 weeks and 6 days and Group 2, comprising prematurely born newborns with GA of 28 weeks or more.

The following data were collected from medical records: gestational age, sex, birth weight (PTM), Apgar score (AS) at 1st and 5th minute and type of respiratory support during echocardiographic examination.

The pwTDI technique was used for echocardiographic measurements, and the values of the maximum systolic velocity of the mitral annulus (s’) and the mitral velocity in early diastole (e’) and late diastole (a’) were determined. Measurements were performed using a 5.5/7.5 MHz cardiac probe and were performed from the apical echocardiographic window (four-chamber view) by adjusting technical parameters according to the specifications described in a previous study [4].

Statistical analysis was performed using appropriate statistical methods and tests. Descriptive data are presented as absolute numbers, frequencies, average values ± two standard deviations, and minimum and maximum values. Data are presented textually, in tables or graphically. The Student’s *t*-test was used to examine the statistical difference between the groups. Pearson’s linear correlation test was used to examine the correlation.

The research was approved by the Ethics Committee of the Institute for Child and Youth Healthcare of Vojvodina in Novi Sad.

## 3. Results

During the study period, 146 prematurely born newborns were hospitalized in the Neonatology Service. The second criterion for inclusion in the study (postnatal age up to 7 days at the time of examination) was met by 97 premature newborns. Among these 97 newborns, the criteria for exclusion from the study were met by 47 premature infants, so the total number of patients included in the study was 50.

The basic characteristics of patients by group are listed in Table 1.

The mean values (± 2SD) of measured pwTDI parameters are listed in Table 2.

When calculated for all 50 subjects together, the Pearson’s linear correlation test showed a strong positive correlation between the values of parameter e’ and GS (*p* = 0, Pearson’s R = 0.88). A positive correlation between the values of parameter e’ and GA was present in subjects from both groups (Group 1 and Group 2), but the correlation among neonates from Group 1 alone was very weak.

**Figure 1:**
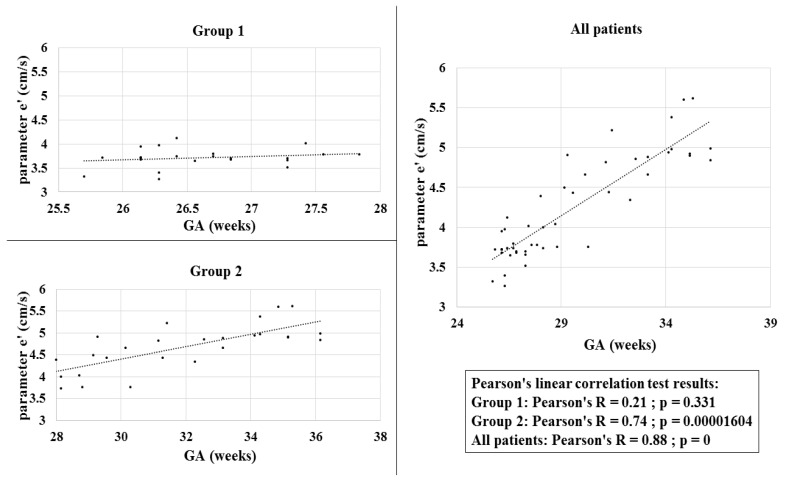
Correlation of parameter e’ and gestational age. Legend: GA—gestational age; parameter e’—velocity of the mitral annulus in early diastole.

Correlations of the values of parameter e’ with GA with the results of Pearson’s linear correlation test (for all subjects included in the study and for each study group) are shown in Figure 1.

**Figure 2:**
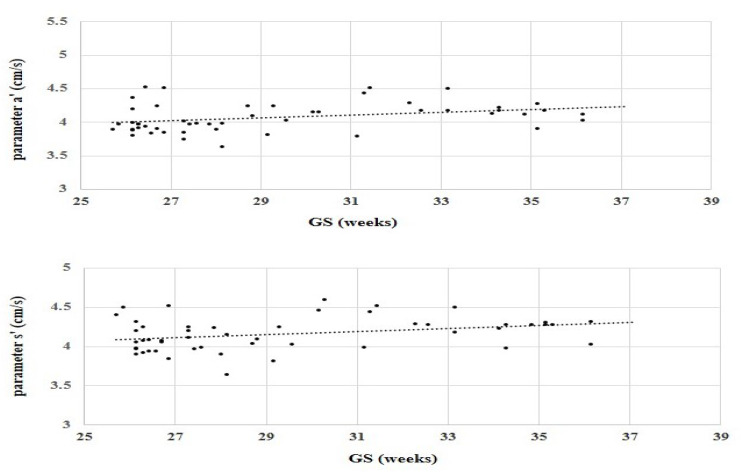
Correlation of parameters a’ and s’ with gestational age for all 50 subjects. Legend: GA—gestational age; parameter a’—maximum velocity of the mitral annulus in late diastole; parameter s’—maximum systolic velocity of the mitral annulus.

When calculated for all 50 subjects, the Pearson’s linear correlation test showed a very weak positive correlation between the values of parameter a’ and GS (*p* < 0.05, Pearson’s R = 0.33) and between the values of parameter s’ and GS (*p* < 0.05, Pearson’s R = 0.31). A weak positive correlation between the values of parameters a’ and s’ with GA was present in subjects from both groups (Group 1 and Group 2), but among neonates from Group 1 alone, the Pearson’s linear correlation test showed almost no correlation (for variable a’, R = 0.08; for variable s’, R = 0.1). Correlations of parameters a’ and s’ with GA for all 50 subjects are shown in Figure 2.

The mean postnatal age at the time of the examination of patients from Group 1 was 3.54 ± 2.28 days, and for subjects in Group 2, it was 3.98 ± 3.40 days. There was no statistically significant difference between postnatal age at the time of examination between subjects from Group 1 and Group 2. (Student *t*-test: *p* = 0.29). Pearson’s linear correlation test (calculated for all 50 subjects) did not show statistically significant association between postnatal age (up to 7 days) and the values of parameters s’, e’ and a’ (*p* = 0.16, Pearson’s R = 0.23; *p* = 0.09, Pearson’s R = 0.21; *p* = 0.10, Pearson’s R = 0.18, respectively).

The total number of children (from both groups) who were on some type of respiratory support (invasive or non-invasive) was 40, of which 21 were on mechanical respiratory support, 12 on nCPAP support and 7 newborns on HFNC. The total number of children who did not required respiratory support was 10. Mean values of echocardiographic parameters are shown in Table 3.

## 4. Discussion

It is well-known that the “immature” myocardium of prematurely born newborns is characterized by low myofibril density; myocytes with simple internal structures and immature and minimally functional organelles (sarcoplasmic reticulum and T tubules); and altered calcium ion turnover, which is crucial for contraction of myofibrils [5,6,7]. The neonatal myocardium has few beta adrenergic receptors and incomplete sympathetic innervation. From birth, the strength of myocardial contraction changes, depending on chronological (not only gestational) age and gradually increasing throughout the neonatal period, primarily due to the increased influx of extracellular calcium. These changes are most intense during the first week of life. Experiments on animal models showed that during the first eight days of life, contractility improved by 45%, and myocardial relaxation improved by 75% [8].

Therefore, it is expected that indicators of systolic and diastolic heart function follow this trend and that their values are influenced by both GA and calendar age. However, the results of our study are only in partial agreement with such claims. In our study, no statistically significant difference was observed between the measured values of echocardiographic parameters obtained using the pwTDI technique between subjects from Group 1 and Group 2 for variables s’ and a’, whereas for variable e ’, a statistically significant difference was found between groups. The measured values of maximal mitral annulus velocity in early diastole (e ’) were statistically significantly higher in preterm infants with higher GA and did not change significantly within the same group during the first week of life. These findings lead to the conclusion that gestational age is among the factors that affect some aspects of left ventricular diastolic function, whereas it has no significant effect on systolic function, and that postnatal age (at least during the first week of life) is not a factor that affects systolic or diastolic performance of the myocardium of the left ventricle.

Similar results were confirmed by a recent study of fetal echocardiography and longitudinal monitoring of fetal heart performance by measuring the same parameters throughout pregnancy, which showed a tendency of increasing mitral e’ velocities with gestational age [9]. Additionally, in a study by Negrin et al., significantly lower values of mitral e’ velocities were obtained in a group of prematurely born newborns with GA younger than 30 weeks compared to a population of newborns with GA of 30 to 36 weeks [4].

Only a few similar studies have been published in the available literature, and the published results are mostly in concordance with those reported in this and previously cited studies, all of which were performed with significant limitations and on relatively small samples [10,11,12]. These limitations highlight the need to additionally examine and determine the reference values of all pwTDI parameters for prematurely born newborns, specifically according to GA, in order to enable a credible interpretation of the findings.

The results of our study show that there was no statistically significant difference between the values of the measured parameters (s’, e’ and a’) in patients who were on some form of respiratory support and those who were not. This finding leads to the conclusion that the use of invasive or non-invasive respiratory support does not significantly affect the performance of the left ventricle. In the available literature, we found only one study in which this correlation was examined in a sample of preterm infants, with findings consistent with the results of our study, although the authors did not explain their finding [3]. Because mechanical ventilation affects the hemodynamic circumstances, predominantly in the pulmonary vascular network, this finding may be expected. Mechanical respiratory support with positive pressure is known to affect left heart function because hemodynamic circumstances in the pulmonary vascular network affect the filling of the left ventricle (preload), indirectly implying that pwTDI indicators of left ventricular systolic and diastolic performance are probably robust to the influence of preload and other factors affected by the use of MV, making them parameters to assess the performance of the left ventricular myocardium, regardless of the status of left ventricular filling and other circumstances related to MV. This is very important because these circumstances may vary significantly in premature infants, influencing and complicating interpretation of echocardiographic findings.

Our study is subject to several limitations. The main disadvantage is the relatively small sample of patients, with insufficient representation of patients of all gestational ages (in particular, the lack of patients with extremely low gestational age), as well as the fact that the study was conducted in only one center. Additionally, the study was performed on a sample of patients who did not have associated diseases that, by different mechanisms, may affect the hemodynamic state and performance of the heart (severe hypoxia, sepsis, etc.), so the findings cannot be applied to patients with such conditions, which are very common in prematurely born newborns [12]. The lack of uniform protocols for the application of this TDI technique in premature infants could also lead to the appearance of technical errors when performing measurements.

Therefore, before applying the echocardiographic technique in routine practice for assessment cardiac function in prematurely born newborns, it is necessary to conduct extensive, multicenter research on a larger and more heterogeneous sample of patients and to determine accurate technical guidelines and valid reference values for all TDI parameters.

## 5. Conclusions

The values of the maximum velocity of the mitral annulus in early diastole (e’) depend on gestational age and are higher in premature neonates of higher gestational age; this correlation is particularly strong in neonates beyond the 28th week of gestation. The values of the maximum velocity of the mitral annulus in systole and late diastole (s’ and a’) are not dependent on gestational age. In preterm infants, echocardiographic indicators of systolic and diastolic left ventricular function measured with pwTDI do not change during the first week of life. In preterm infants, the use of invasive or non-invasive respiratory support does not affect the values of pwTDI indicators of systolic and diastolic left ventricular function.

## Figures and Tables

**Table 1:** Characteristics of the patients included in the study.

	Group 1	Group 2	All Subjects
Number of patients	24	26	50
Mean postnatal age in days ± 2SD (min–max)	3.56 ± 1.14 (0–6)	4.22 ± 0.98 (0–7)	3.42 ± 1.06 (0–7)
Mean GA (weeks) ± 2SD	26.60 ± 1.16	31.94 ± 5.47	29.38 ± 6.71
Gender (male/female)	14/10	12/14	26/24
Mean body weight (g) ± 2SD	843.34 ± 96.22	1348.71 ± 42,584	1354.77 ± 62,372
Mean AS at 1st minute ± 2SD	3.48 ± 2.58	6.12 ± 3.44	4.62 ± 4.22
Mean AS at 5th minute ± 2SD	4.88 ± 2.26	6.96 ± 3.86	5.84 ± 3.12
Number of patients on MV, nCPAP or HFNC (yes/no)	22/2	18/8	40/10

Legend: GA—gestational age, AS—Apgar score, MV—mechanical ventilation, nCPAP—nasal CPAP, HFNC—high-flow nasal cannula, SD—standard deviation.

**Table 2:** Mean values of echocardiographic parameters obtained using the pwTDI technique.

pwTDI Parameter	Group 1	Group 2	*p* Value	All Patients
s’ (cm/s) ± 2SD	4.06 ± 0.78	4.18 ± 1.22	0.42	4.10 ± 0.32
e’ (cm/s) ± 2SD	3.71 ± 0.40	4.68 ± 1.04	0.0001	4.21 ± 0.26
a’ (cm/s) ± 2SD	3.98 ± 1.06	4.12 ± 0.94	0.31	4.07 ± 1.44
ratio e’/a’ ± 2SD	0.93 ± 0.13	1.11 ± 0.23	*p* < 0.0000	1.02 ± 0.26

Legend: s’—maximum systolic velocity of the mitral annulus, e’—velocity of the mitral annulus in early diastole, a’—velocity of the mitral annulus in late diastole, *p* value (Student’s *t* test).

**Table 3:** Mean values of echocardiographic parameters obtained using the pwTDI technique in neonates on respiratory support and without respiratory support.

pwTDI Parameter	Respiratory Support	Without Respiratory Support	*p* Value
s’ (cm/s) ± 2SD	4.08 ± 0.66	4.10 ± 0.38	0.857
e’ (cm/s) ± 2SD	3.82 ± 0.54	3.93 ± 0.74	0.32
a’ (cm s) ± 2SD	3.92 ± 0.96	3.84 ± 1.04	0.08

Legend: s’—maximum systolic velocity of the mitral annulus, e’—velocity of the mitral annulus in early diastole, a’—velocity of the mitral annulus in late diastole, *p* value (Student’s *t* test).

## Data Availability

Not applicable.
